# High-Resolution Positional Tracking for Long-Term Analysis of *Drosophila* Sleep and Locomotion Using the “Tracker” Program

**DOI:** 10.1371/journal.pone.0037250

**Published:** 2012-05-15

**Authors:** Nathan Donelson, Eugene Z. Kim, Justin B. Slawson, Christopher G. Vecsey, Robert Huber, Leslie C. Griffith

**Affiliations:** 1 Department of Biology, National Center for Behavioral Genomics and Volen Center for Complex Systems, Brandeis University, Waltham, Massachusetts, United States of America; 2 Department of Biological Sciences, JP Scott Center for Neuroscience, Mind, & Behavior, Bowling Green State University, Bowling Green, Ohio, United States of America; Queensland Brain Institute, Australia

## Abstract

*Drosophila melanogaster* has been used for decades in the study of circadian behavior, and more recently has become a popular model for the study of sleep. The classic method for monitoring fly activity involves counting the number of infrared beam crosses in individual small glass tubes. Incident recording methods such as this can measure gross locomotor activity, but they are unable to provide details about where the fly is located in space and do not detect small movements (i.e. anything less than half the enclosure size), which could lead to an overestimation of sleep and an inaccurate report of the behavior of the fly. This is especially problematic if the fly is awake, but is not moving distances that span the enclosure. Similarly, locomotor deficiencies could be incorrectly classified as sleep phenotypes. To address these issues, we have developed a locomotor tracking technique (the “Tracker” program) that records the exact location of a fly in real time. This allows for the detection of very small movements at any location within the tube. In addition to circadian locomotor activity, we are able to collect other information, such as distance, speed, food proximity, place preference, and multiple additional parameters that relate to sleep structure. Direct comparisons of incident recording and our motion tracking application using wild type and locomotor-deficient (*CASK-β* null*)* flies show that the increased temporal resolution in the data from the Tracker program can greatly affect the interpretation of the state of the fly. This is especially evident when a particular condition or genotype has strong effects on the behavior, and can provide a wealth of information previously unavailable to the investigator. The interaction of sleep with other behaviors can also be assessed directly in many cases with this method.

## Introduction

*Drosophila* sleep and locomotor patterns provide important models for studying the molecular and genetic bases for complex behavior. As in mammals, circadian rhythms in *Drosophila* are endogenous and robust [1], [2]. *Drosophila* sleep behavior also shows many of the same properties as mammalian sleep: it can be disrupted by environmental stimuli and psychostimulants, its amount and structure varies in an age-dependent manner, and importantly, it is homeostatically regulated [3]–[5]. All of these features can be monitored through assessment of locomotor and circadian activity. Many of the genes identified in mammals and other animals as being important for sleep regulation have close homologs in *Drosophila*, which has the added benefit of highly tractable genetics [6], [7]. Thus, these qualities make *Drosophila* an important resource in understanding the mechanics of sleep.

**Figure 1:**
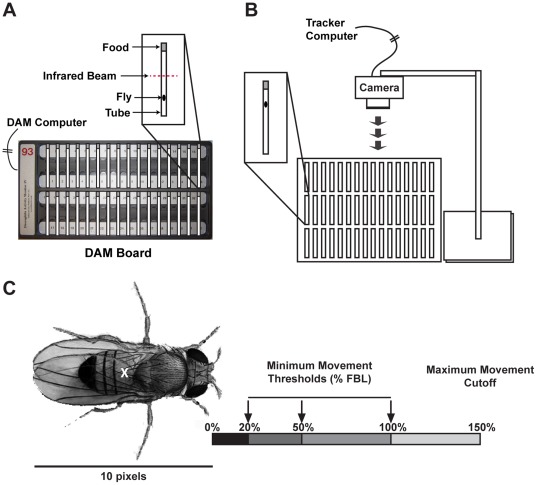
Data recording techniques. Sleep/Locomotor data can be recorded using two fundamentally different techniques. (A) DAM boards use infrared beams to record the number of times a fly interrupts the beam. (B) Tracking software uses a video camera to record the location of each fly as coordinate data at 1 Hz intervals. (C) Because the size of the fly varies for different focal distances, it was necessary to standardize data across experiments by measuring movement based on the overall length of the fly in pixels. For these experiments, the flies were ∼10 pixels long. Therefore, the Fly Body Length (FBL) was 10 pixels. A minimum threshold for movement could then be applied to Tracker data to standardize movement across different lighting conditions to determine at what point movement occurred. By restricting the data to a minimum movement value, the center of the fly (X) had to move at least that Euclidean distance before it was scored as movement. Three minimum thresholds compared in this study (20%, 50% or 100% FBL) are shown. The Maximum cutoff value for movement restricted the data to distances that were actually attainable. Flies could physically only move so far within the tube (never greater than 150% FBL in our experiments for a 1 sec time window). Both the minimum and maximum cutoffs function to control for random data error.

Locomotor and sleep research, amongst others, has relied on incident or event recording to measure behavior for decades. Due to technological limitations, this method was for many years the only practical option to capture behavioral data over the course of many days. In the most widely used system for flies, the *Drosophila* activity monitoring (DAM) (Trikinetics, Waltham, MA) system, animals are placed individually into 65×5×5 mm glass tubes that are bisected with an infrared beam (Figure 1A). Whenever a fly breaks the beam, an activity event is tallied for later analysis. In this manner, the beam crosses provide a very general picture of the locomotor activity of the fly, and data collected in 1–30 min bins have long been used to depict circadian rhythms. This system has been adapted to measure sleep by capitalizing on the observation that animals which are immobile for more than 5 min show behaviors consistent with a sleep state, including altered posture, increased arousal threshold, and altered central nervous system activity [3], [4], [8], [9]. For measuring sleep, incident data are collected in 1 min bins and analyzed for periods of ≥5 min with no event. This method has become the standard of the field, and has led to many new insights into fly sleep behavior.

While gross locomotor and sleep patterns can be observed with beam disruptions, the only time that the true activity of the fly is known is at the exact instant that the beam is crossed. Accurate measurements of a phenotype could be lost in the analysis with this type of data. Locomotor-deficient flies are expected to break the beam less frequently than wild type, but the severity of the locomotor phenotype could be underestimated. Poorly moving flies may not travel the whole tube length, and DAM does not account for flies that spend large amounts of time at one end of the tube eating, grooming, or spinning in circles. These behaviors could be misidentified as sleep. In both flies and humans it is clear that many sleep and locomotor disorders are complex, with the effects on one behavior not mutually exclusive from the other (e.g. many neurodegenerative diseases) [10]–[12]. A comprehensive analysis of these conditions requires the development of alternative methods for quantifying sleep and locomotor behavior over extended time periods.

As computer processing has become more powerful and camera systems have become smaller and no longer require discs or tapes, video-based computerized tracking has become a practical technology. Recently, several fly labs have developed specialized software for video-based data capture [13]–[17]. Instead of video recording entire experiments, which are large and unmanageable for storage and processing, Zimmerman et al. [18] developed a system that captures a single image every 5 sec, and once finished, uses contrast comparisons between sequential images to quantify changes in fly position. Although this reduces video file sizes considerably, each experiment still requires many GB of storage space, and because the fly is only monitored every 5 sec, this technique lacks spatial and temporal resolution about the detailed behavior of the animal.

The present study utilizes a contrast-based, computerized video tracking system that records text-based coordinates in real time at high temporal resolution (Figure 1B) without the need to store video of images. The tracking application used for this study is capable of tracking many isolated flies over many days, following a circadian light/dark schedule, without interruption, at a temporal resolution of 1 sec or less. This system creates files that are relatively small for location data (1 GB for 14 days, 100 flies), and is capable of providing spatial information that event recorders cannot. This software package is based on the image processing media module of the JavaGrinders library (public-domain under the GNU license at iEthology.com). Here, we report a direct comparison between incident recording data (DAM) and data from our video-based tracking system (Tracker). We demonstrate how beam-cross data can, for some genotypes, misrepresent the true behavior of an animal and miss important aspects of a phenotype. We also show that video tracking can be analyzed to extract information germane to behavior (distance moved over time and place preference during sleep and wake, for example) that beam-cross data are unable to provide.

## Materials and Methods

### Fly Handling

We conducted two separate experiments for this paper, the first using *Canton S* (*CS*) wild type males (N = 8) and the second using females from both a *CASK-β* null (*CASK^P18^*) (DAM *CASK-β* N = 30, Track *CASK-β* N = 30) and a precise excision strain as a wild type genetic control line (*CASK* ^P33^) (DAM Control N = 30, Track Control N = 29) [19]. All flies were raised on a cornmeal-sucrose-agar food in a 25°C incubator with a 12-hr Light/Dark cycle and were 3–5 days old at the start of each experiment. Flies were loaded under CO_2_ anesthesia into individual glass tubes. Each tube contained an agar/sucrose food plug sufficient to sustain the fly for the duration of the experiment. The tubes were sealed with parafilm at both ends to allow for the fly to be tracked all the way to the end of the tube without visual obstruction. For tracking, the tubes were taped to a piece of white office paper, providing a high visual contrast field against the dark fly and transparent glass tube (Figure 1B). The paper was positioned inside of an incubator under a USB video camera (Logitech, Quickcam for Notebooks). A red compact fluorescent bulb and red LEDs, emitting a wavelength of light not detected well by the fly visual system [4] and incapable of entraining *per^01^* flies (N.D. unpublished observations), were placed into the incubator to provide enough light for the camera to maintain an image when the white lights were off during the night. While Gilestro and Cirelli [20] used infrared (IR) lighting to follow flies in the dark, we achieved better contrast and illumination with red LEDs while also avoiding the excessive heat that we found was generated from the IR emitters. Flies were also loaded into DAM boards as previously described for collecting beam-cross data [21], and run in parallel to the Tracker flies in the same incubator. Data were collected following three days of light∶dark (LD) entrainment to a 12 h∶12 h cycle.

### Tracking Flies

The Tracker application is a Java-based program that uses image subtraction to identify the location of a dark object on a light background or a light object on a dark background. The program first captures a reference image of the tubes without the flies. After adding the flies to the tube, video frames are obtained from a live stream at scheduled times and compared with the reference. The flies represented by the areas of a given contrast and size, are characterized as bounding polygon at a given threshold. The area's center pixel is recorded as a textual X/Y coordinate at a user-defined time interval. Many independent objects (track jobs) can be run simultaneously by defining multiple, specific locations for the Tracker program to look for contrast differences. Each track job must be a discrete, non-overlapping area that contains a single fly. The number of track jobs that can be simultaneously recorded depends on how many objects can fit into the visual field of the camera. The temporal resolution for the data capture can be adjusted, ranging from 30 frames per second to much longer intervals. For these experiments, the coordinates for all of the flies were saved at 1 Hz into a single text file. Thus, this method of data capture eliminates the need for stored video or pictures, as well as the need for post-processing of video and pictures. Although no video is saved to a file, the Tracker program does display the live video while an experiment is running, allowing the experimenter to monitor (or score) the flies at any time. All Tracker program application files, explicit operating instructions, and analysis scripts are available for download (http://www.bio.brandeis.edu/tracker). For comparative purposes, we recorded 20 minutes of video while also running the Tracker. The experiment was conducted at ZT16 using 5 CS females raised as described above. The video was hand-scored for detectable body movement at the same focal distance as the tracker, and the movement scores were compared with data produced by the tracker.

### Statistical Analyses

**Figure 2:**
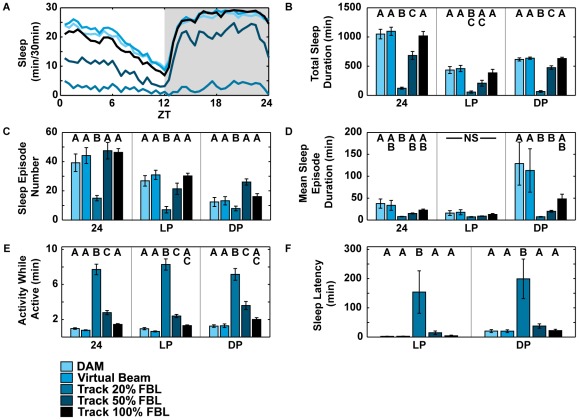
Data capture comparison for *CS* flies shows how restricting Tracker output conforms location data to DAM-like outputs. Sleep parameters from DAM and Tracker program analysis for *CS* wild type flies (N = 8). Data from 3–5 day old male flies were collected for three consecutive days in a 25**°**C incubator with 12-hr LD cycle. Same letters indicate no significant difference between methods using Tukey HSD (*P*<0.05). NS = No Significant difference. Individual statistical tests were performed on each time comparison group (24, LP, DP). The order of letters represents the order of the analysis: DAM, Virtual Beam, Track 20%, Track 50%, Track 100% of the Fly Body Length (FBL). (A) Sleep profile for flies averaged for three days. (B, C, D, E, F) DAM data matches the Virtual Beam for all comparisons. Track 100% FBL matched DAM for all comparisons as well, but was also not significantly different from 50% FBL in many comparisons. 20% FBL was always significantly different from DAM. The 50% FBL showed significant differences from DAM in many comparisons, while still showing similarity, indicating that this resolution was most effective at capturing both DAM-insensitive and biologically meaningful locomotion.

**Figure 3:**
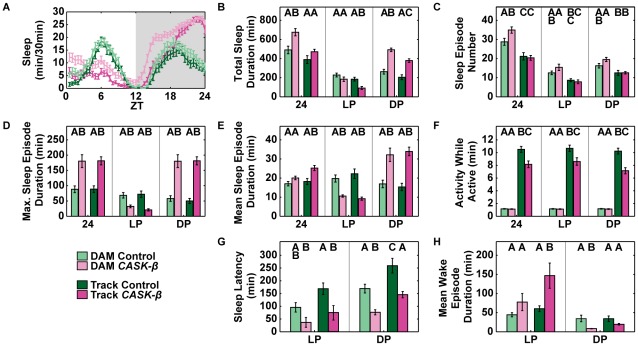
Sleep data capture comparison for DAM and Tracker sleep data. Sleep parameters from DAM and Tracker analysis for flies averaged for three days, DAM Control (N = 30), DAM *CASK-β* (N = 30), Track Control (N = 29), Track *CASK-β* (N = 30). Data from 3–5 day old female flies were collected for three consecutive days in a 25**°**C incubator with 12 hr LD cycle. Same letters indicate no significant difference between methods using Tukey HSD (*P*<0.05). A separate Tukey test was run for each time comparison (24, LP, and DP). (A) Sleep profiles show higher sleep in the mutant line recorded by DAM. (B) Total sleep duration shows lower sleep at night and higher sleep during the night in the Tracker data as compared to control. (C) The number of sleep episodes was not significantly different in Tracker data. (D) Maximum sleep episode duration did not differ across methods, but was significantly different between genotypes. (E) DAM did not identify a difference in Mean sleep episode duration total. The Tracker program detected a difference at 24, LP, and DP. (F) DAM was not able to detect the locomotor deficiency in the *CASK-β* mutant. (G) Tracker data shows a greater latency difference between the genotypes in LP and DP. (H) The DAM and Tracker data show opposing results in mean wake duration depending on the time of day. DAM shows no difference in the LP, while Tracker data shows no difference in DP.

Sleep data were analyzed using JMP 9 statistical software (SAS Institute). For data shown in Figure 2, a one-way ANOVA with analysis type (DAM, Virtual Beam, and Tracker using 20%, 50%, or 100% fly body length) as a factor was run for each analysis period (24, LP, and DP) (Table 1). For data shown in Figure 3, a one-way ANOVA was used with genotype and analysis type combined into a single factor (DAM Control, DAM *CASK-β*, Track Control, and Track *CASK-β*) for each analysis period (24, LP, and DP) (Table 2). Individual Tukey HSD post-hoc tests were performed based on the ANOVA results to determine which pairs were significantly different. All comparisons are marked by letters (A, B, C, or D) in Figures 2 and 3. Bars not matched by the same letter within an analysis period are significantly different. T-tests were used to compare movement distances at ZT12 and ZT24.

**Table 1:** List of ANOVA results for Figure 2.

Sleep Measurement	Time Period	F ratio	P value
Total Sleep Duration	24	36.8986	<0.0001
	LP	11.5115	<0.0001
	DP	105.8450	<0.0001
Sleep Episode Number	24	8.4188	<0.0001
	LP	10.4289	<0.0001
	DP	8.0402	0.0002
Mean Sleep Episode Duration	24	3.0857	0.0306
	LP	1.4651	0.2375
	DP	3.0124	0.0335
Activity while Active	24	95.8137	<0.0001
	LP	107.9081	<0.0001
	DP	40.1201	<0.0001
Sleep Latency	LP	4.1376	0.0087
	DP	6.4984	0.0007

The ANOVA table shows that at there was a significant difference for each analysis type (DAM, Virtual Beam, 20%, 50%, and 100% FBL) at each time period (24, LP, or DP) except for Mean Sleep Episode Duration LP. Results from individual Tukey pairwise comparisons are shown in Figure 2. All analyses except Mean Sleep Episode Duration during LP were significant at DF_(4,30)_, *P*<0.05, N = 7 for all analysis types.

**Table 2:** List of ANOVA results for Figure 3.

Sleep Measurement	Time Period	F ratio	P value
Total Sleep Duration	24	10.7667	<0.0001
	LP	8.6607	<0.0001
	DP	33.7691	<0.0001
Sleep Episode Number	24	16.9581	<0.0001
	LP	9.1018	<0.0001
	DP	9.6764	<0.0001
Max. Sleep Episode Duration	24	13.1055	<0.0001
	LP	11.9278	<0.0001
	DP	27.4459	<0.0001
Mean Sleep Episode Duration	24	8.1198	<0.0001
	LP	16.1862	<0.0001
	DP	15.3452	<0.0001
Activity While Active	24	189.1337	<0.0001
	LP	164.6881	<0.0001
	DP	182.3627	<0.0001
Sleep Latency	LP	6.0076	0.0008
	DP	16.6363	<0.0001
Mean Wake Episode Duration	LP	4.8440	0.0033
	DP	5.0937	0.0024

The ANOVA table shows that at least one factor was significantly different for each sleep parameter. Results from individual Tukey pairwise comparisons are shown in Figure 3. All analyses were significant at DF_(3,115)_, *P*<0.05, DAM Control (N = 30), DAM *CASK-β* (N = 30), Track Control (N = 29), Track *CASK-β* (N = 30).

## Results

To directly compare traditional DAM system data collection with video tracking, wild type male *CS* flies were placed into a DAM board that was positioned under the video camera for tracking. This allowed beam-cross and tracking data to be simultaneously collected on the same population of flies.

### Data resolution changes interpretation

The coordinates of the Tracker program output were transformed into DAM-like data using custom scripts with Matlab R2011a (Mathworks, Natick, MA). For each individual fly, we converted the difference between sequential data points into a binary code to determine if the fly had moved during that second or not (1 = move, 0 = no move). We found that this was necessary to make sensitivity adjustments to the Tracker data. At the focal distance of this experiment, the fly was ∼10 pixels long. A 1-pixel movement criterion for sequential data points was found to be too sensitive, making the flies appear to be moving continually. A 20-pixel movement criterion was too insensitive – flies appeared completely immobile because they never moved more than 15 pixels in 1 second (i.e. 20 pixels/sec exceeded their maximum speed at this focal distance). Differences in white light and red light can also alter how a camera sees an image. Dim light does not have as crisp a focus as bright light. The lack of crisp focus means that there is a possibility of detecting small quality-related differences in sequential images that could be misinterpreted as movement. By setting a minimum distance that the fly had to move, we could standardize movement under varying conditions. For this experiment, Tracker data files were made using minimum movement thresholds of 2, 5, or 10 pixels, from the same data set, to compare to standard DAM recorded data. This represented roughly 20%, 50%, and 100% of the fly body length (FBL), or the minimum distance that a fly had to move in any direction before movement was detected (Figure 1C). Since focal distance can vary between experiments and camera setups, we have used FBL as a metric to allow direct comparisons between data sets.

The transformed Tracker data were processed into 1 and 30-min bins using a format that was identical to standard DAM files. The three days of beam-cross data collected from the actual DAM board was formatted using DAMFileScan102X (Trikinetics, Waltham, MA) into 1 and 30 min bins. We did one additional control manipulation of the Tracker data to transform it into ‘virtual’ beam-cross data. We identified the X/Y location of the infrared beam in the DAM board and created a ‘virtual beam’ in the Tracker data at that location. The fly coordinates were converted into a binary code and formatted into DAM-like files so that an activity event was recorded whenever the fly bisected the ‘virtual beam’ (1 = crossed, 0 = not crossed). If the Tracker program recorded data properly, Virtual Beam data should be exactly the same as DAM data.

We analyzed the DAM, Tracker (20%, 50%, 100% FBL), and Virtual Beam data using traditional circadian and sleep analysis software [22] and new Matlab-based analyses developed within the Griffith lab. The experimental days were averaged together, and sleep data were plotted as a line plot for the amount of sleep per 30 min (Figure 2A). Data were also analyzed for total sleep, latency to sleep onset, mean sleep episode duration, number of sleep episodes, and how active the flies were when they were awake (Figures 2B–F).

Tracker-recorded data are able to replicate the sleep behavior results of DAM board-recorded data for wild type flies. Figure 2 compares DAM, Virtual Beam, and 20%, 50%, or 100% FBL Tracker data using traditional sleep plot analyses. The differences between the DAM data and Virtual Beam data were not significant for any measured parameter. Thus, at a resolution equal to DAM, the Tracker data produced identical results. Every data point reported at higher resolutions than Virtual Beam (20%, 50%, 100% FBL) was activity that DAM beam-cross data collection had missed.

Varying the threshold criterion for movement shows that the sensitivity of the Tracker program can greatly alter reported sleep values. At 20% FBL resolution, flies are almost always scored as active (Figures 2A, 2E, and Table 1) and sleep latency values are significantly longer than for all other data sets (Figure 2F), suggesting that the amount of “movement” at this resolution is likely to be either a capture artifact (e.g. small changes in the shape of the fly due to focus differences in white and red light, or camera noise) or to reflect small movements that are not altered by sleep/wake state (e.g. postural change). This was verified by visual scoring, which found that a 20% FBL scored flies as active ∼89% more than by hand-scoring.

The 50% and 100% FBL thresholds produced an output that was more similar to the DAM data. The 100% FBL resolution was the closest match to the beam-cross data, and not significantly different from DAM or Virtual Beam for any parameter. 100% FBL also was not significantly different from 50% FBL for any of the 12 h measures except dark period total sleep duration (F_(4,30)_ = 105.85, *P*<0.0001). More differences were seen between 50% and 100% FBL when data was averaged over 24 h, as seen in both lower total sleep (Figure 2B) and a higher amount of movement when the flies were active (Figure 2E) using the 50% calculation. Overall, we find that the Tracker program detects higher activity levels and shorter sleep episode bout durations, indicating that DAM generally overestimates sleep. Interestingly, this is most significant during the dark period (Figures 2C and 2F), and may indicate that flies sleep in a less consolidated manner at night than previously thought.

We favor using the 50% FBL threshold since it does not require large positional changes by the fly to register as a movement, while still capturing the architecture of fly sleep. Hand-scoring also showed the best correlation with 50% FBL measurements of activity. The 50% FBL detected ∼12% more movements than hand-scoring, indicating that the observer could not detect the exact onset/termination of movement or that the movement was of too short duration/distance to visually detect. The 100% FBL detected only 19% of the fly movement detectable by hand-scoring, suggesting that this high threshold (and by extension, the prevalent DAM scoring system) missed a large portion of fly movements.

### Tracker and DAM analysis diverge more significantly when experimental animals have complex sleep phenotypes

The generally good agreement between DAM and Tracker in the 50% to 100% FBL range of threshold was not completely uniform across all sleep parameters, even for wild type flies. The failure of DAM to accurately measure specific parameters could become even more pronounced if a particular parameter, such as sleep episode duration, is highly variable between genotypes. To test this, we conducted an experiment to assess the ability of the DAM system and the Tracker program to measure sleep using a locomotor-deficient fly strain and its genetic control. *CASK-β* null mutants have been previously shown to display a robust locomotor phenotype [19], and thus provided a good test case for comparing the two systems. Concurrent DAM and Tracker (50% FBL) data for *CASK-β* null and control flies were collected and analyzed as above.

For the precise excision control line (green bars in Figure 3), DAM and Tracker data 12 h parameters were statistically indistinguishable except for activity (Figure 3F and Table 2) and dark period latency (Figure 3G), consistent with the agreement between the two methods seen with *CS* wild type. The two methods differed substantially, however, when used to assess the *CASK-β* mutant (pink bars in Figure 3). The DAM system recorded significantly more sleep episodes and overall higher amounts of sleep in the mutant (Figures 3A–C). Total sleep duration was particularly interesting, because the DAM data suggested that a nighttime difference alone contributed to the higher sleep levels in the mutant flies. In contrast, the Tracker data detected a sleep difference in both the day (low) and the night (high), and no overall difference in total sleep, suggesting that this mutant redistributes its sleep. This was also seen clearly in the sleep plot where DAM appeared to overestimate early morning and early evening sleep (Figure 3A). The sensitivity of the Tracker program detects small movements missed by DAM, as well as circadian aspects of the *CASK-β* locomotor defect (see below). The results also show that the Tracker data detected sleep differences between the two genotypes that DAM data alone did not (Figures 3B, 3G, and 3H). Most importantly, the standard DAM assessment of general locomotion (Figure 3F) did not detect a locomotor deficit in the mutant, even though this phenotype can be observed by eye [19].

### The Tracker program can also assay locomotor parameters at a high resolution

**Figure 4:**
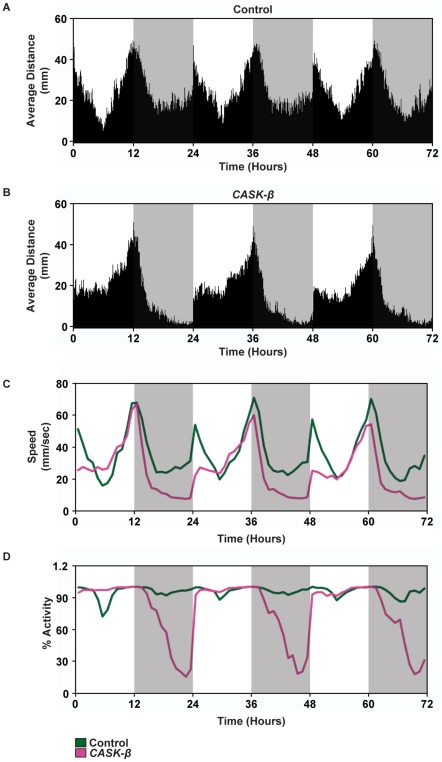
Control flies move much greater distances than *CASK-β* mutant flies and have a more dynamic locomotor pattern. Distance was averaged for each genotype and binned into minutes. (A) Female wild type control flies (Control, N = 29) had stereotypic locomotion across the day. (B) Female *CASK-β* mutant flies (*CASK-β*, N = 30) had a locomotor profile that was different from control. Nighttime movements were very low, with no morning light anticipation. (C) Speed plot for each genotype. (Green = control, Pink = *CASK-β*). The speed of the mutant fly followed closely with control only in the hours leading up to lights out. (D) All flies did not behave exactly the same all the time. Behavioral state was intrinsic to the individual. % Activity shows that control flies alternated their sleep/wake activity so that at least one fly was active all throughout the day. There was an overall sleep rhythm, but some individuals were awake while others were asleep. By contrast, there were long periods where all *CASK-β* mutant flies were not moving at night.

The Tracker program gathers data about fly movement patterns that are impossible to obtain through beam-cross measurement. We analyzed the Tracker data as its original coordinate data to extract locomotor information. This new level of detail shows how the flies moved over the course of the experiment, how different time points compare statistically, and allows for simultaneous comparisons between sleep and locomotion using a single data set. For this experiment, we calculated the average distance the flies moved during specific times of the experiment, and provide a sample of other descriptive statistics for locomotion (Figure 4, Table 3).

**Table 3:** Examples of descriptive statistics that can be gleaned from Tracker data that could not be determined from standard beam cross data (Track *CASK-β* N = 30, Track Control N = 29).

	Track Control	Track *CASK-β*
Total Distance Per Day (mm)	Day1 – 29357.20	Day1 – 20867.02
	Day2 – 30261.55	Day2 – 19725.89
	Day3 – 30104.26	Day3 – 17901.91
Total Distance (mm)	86,889.21±15,372	58,494.82±10,259
Average Distance (mm) Hour 12	2214.96±113.17	2060.77±113.12
Average Distance (mm) Hour 24	1137.63±47.72	89.90±13.14

The control flies' movement was markedly different from the *CASK-β* mutant flies at all points of the day except for immediately before lights out (ZT11–12). Over the course of three days, the control flies on average moved 86,889±15,372 mm, or almost 90 m. By comparison, the mutant flies moved only 67% of the distance of the control, moving 58,494±10,259 mm. This difference in distance was dependent upon the time of day. The fact that there was no significant difference in how far the flies moved at ZT12 (t_(4)_ = 2.77, P = 0.39) suggest a strong circadian difference between the locomotor drive of the mutant and its control. Additionally, unlike the controls, the mutant flies did not anticipate the lights turning on in the morning, and barely moved compared with controls before lights on at ZT24 (t_(2.3)_ = 2.77, *P*<0.01). This variability in the distance travelled at different times of day for the mutant is problematic for DAM because it records movement indirectly. The locomotion of the mutant flies is unlinked to the sleep/wake cycle. This is evident by comparing sleep and distance travelled at ZT18 (Figure 3A). Both fly lines have the same sleep state at this time point (Figure 3A), but control flies are moving much more than *CASK-β* mutants (Figure 4B).

The speed of the flies directly correlated with the distance they traveled (Figure 4C). As the speed of the flies increased, so too did the distance moved. When looking at the amount of time the flies were active (Figure 4D), there were distinct differences between the two genotypes that directly relate to sleep, distance, and speed. Control flies had a very consistent population activity level over the course of the entire experiment. There were very few instances when all of the control flies were inactive at the same time. The mutant flies had a clear pattern of activity loss at night, when almost none of the flies were moving for long periods of time. This data indicates that, in addition to abnormal sleep rhythms, the mutant flies also lack the same kind of persistent activity bouts observed in the wild type throughout the night. The circadian nature of the locomotor defect in the *CASK-β* mutant is clearly shown by this direct analysis of activity.

### Tracker data can also be used to assess place preference

**Figure 5:**
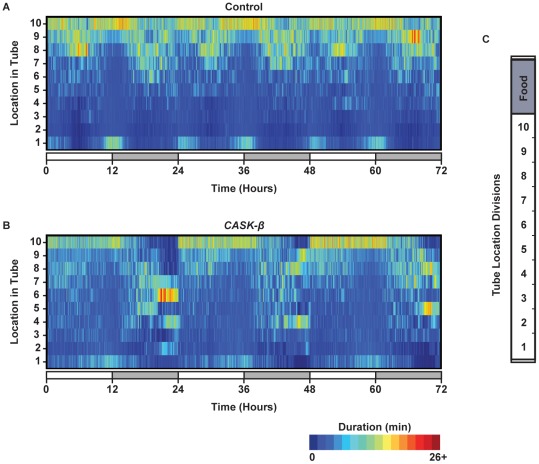
Location plots show that *CASK-β* mutant flies have altered place preference compared with controls. Location plots show the place preference of the wild type control and *CASK-β* mutant flies over the course of the experiment. Place preference was determined by recording the location of the flies within their tubes, and calculating the amount of time spent in each location as a function of time of day. Here, the fly locations were binned every minute of the experiment. (A) Female wild type control (Control, N = 29) flies have a periodic rhythmicity to place preference. (B) The female *CASK-β* mutants (*CASK-β*, N = 30) have altered place preference compared with control flies, and show longer durations spent in the center of the tube. Color intensity indicates longer dwell time for that location. Each individual vertical segment represents 1 min of the experiment. The tube was also divided into 10 equal sized sections (C). The food was always located just above section 10. White color in the time bar = daytime. Grey color in the time bar = night.

Tracker program coordinate data is a record of the spatial location of the fly across time. We used the spatial information to identify where flies were in the tubes and for how long they were there. Figure 5 shows an overall picture for where the flies were over 3 days of observation in LD. The flies had location preferences at specific times of the day and night, and these preferences were specific to genotype. The control flies had a very periodic preference pattern for tube location and a characteristic dwell time. Controls also preferred to be in the quarter of the tube closest to the food during the day (Figure 5A). The amount of time spent in section 1 (farthest from the food) peaked when the lights switched, probably reflecting both a startle response and the normal circadian morning and evening peaks of activity. This correlated well with the distance and sleep data. The flies did not spend much time idle in the half of the tube farthest from the food, particularly during the daytime siesta (ZT6).

The location preference of *CASK-β* mutant flies was starkly different from the control. In general, the mutant flies did not have the same rhythmic location consolidation, particularly for locations near the food (section 10). They occupied the entire length of the tube, as noted by the lighter color blue throughout the middle of the tube as compared with controls. This middle tube lingering may be expected given the mutant's locomotor problems [19]. The *CASK-β* mutant flies also did not have preference peaks at location 1 or 10 during the night. Rather, they spent most of the night within the middle of the tube. Unlike the control flies that preferred the half of the tube near the food at night, the mutant flies seemed to avoid the food at this time. The location preference changed as soon as the lights turned on at ZT0, taking them closer to the food for the remainder of the day. This difference in patrolling behavior and place preference would be expected to complicate beam break comparisons between these two lines and bring into question the ability of DAM to adequately measure sleep and locomotion in fly lines with complex phenotypes.

**Figure 6:**
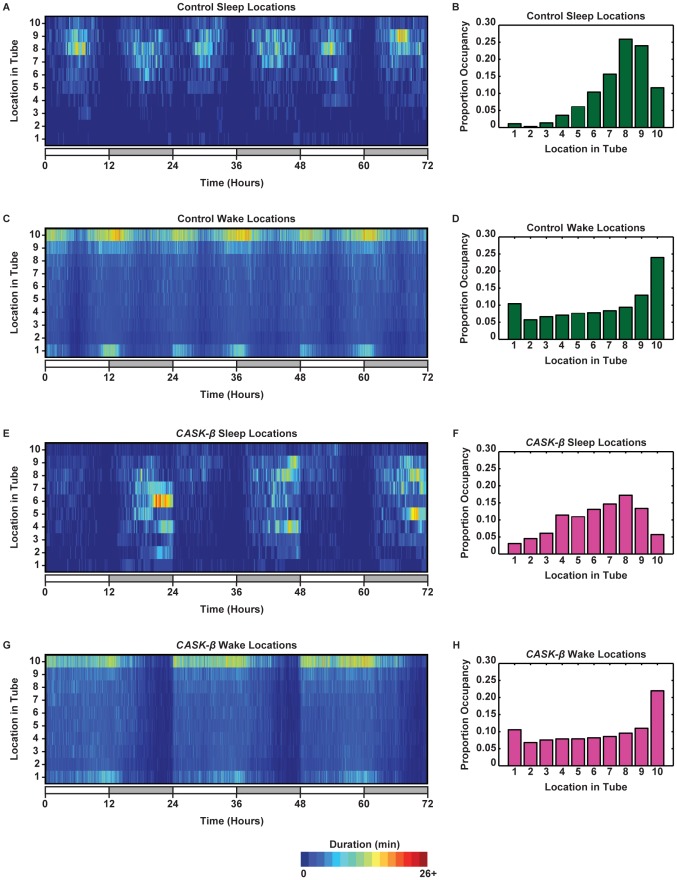
*CASK-β* mutant flies have different location preferences from controls when both asleep and awake. Location and Proportion of Occupancy plots show where the wild type control (Control, N = 29) and *CASK-β* mutant (*CASK-β*, N = 30) flies were when they were awake and asleep. (A, B) Female wild type control flies sleep near the food during siesta and at night and rarely venture to the half of the tube away from the food. (C) Wild type control flies show preference for being at the food when awake. The flies moved throughout the entire length of the tube, pausing at either end. This location preference coincides with peak activity times of day. (D) When awake, Control flies spent ∼24% of their time at the food. The rest of their waking time was dispersed throughout the tube. (E, F) Female *CASK-β* mutants sleep at different times and locations than the controls and do not have siesta. When the *CASK-β* mutants did sleep, they were dispersed throughout sections 4–9 of the tube. (G, H) When awake, the *CASK-β* mutants showed little location consolidation across the day as compared to control and spent the majority of their waking time at the food (∼22%). The rest of their time was spent dispersed throughout the rest of the tube, with some preference for the far end.

We investigated location and preference in more detail by plotting the locations of the flies when they were asleep and awake. Figure 6 shows locations over 3 days in 1 min bins and gives a plot of proportion of time occupancy of each bin collapsed for all three days. The Control flies preferred locations near the food when asleep (Figures 6A and 6B). Surprisingly, the Control flies slept at the same location whether during siesta or at night, indicating that location preference is not dependent upon lighting condition and time. When awake, the Control flies had their highest preference for being right at the food (Figure 6C, D). The Control flies were most active right before light changes (Figures 4A and 6C). The locations that the Control flies occupied during this peak activity indicates that the behavior of the flies follows a repeating pattern of quickly moving through the center of the tube, pausing to eat at the food, passing through the center again, briefly pausing at the cap end (perhaps exploring or turning), and returning to the food. In general, preference for a particular location decreased the further away the fly was from the food (Figure 6D).

The *CASK-β* mutant flies had different sleep and wake location preferences. Notably, the *CASK-β* mutant flies didn't have a mid-day siesta or morning anticipation (Figures 6E and 6G). The *CASK-β* mutant flies also did not have the same location preference as the Control flies. When sleeping, the *CASK-β* mutant flies had a location preference that was further away from the food, more central in the tube, and more spread out (Figures 6E and 6F). When awake, the *CASK-β* mutant flies showed the same overall location preference as the Control flies (Figures 6D and 6H), but the temporal pattern for location was noticeably different (Figures 6C and 6G). The *CASK-β* mutant flies did not have the same consolidation of their time at the food location. Rather, their preference for location 10 was more diffuse throughout the entire time they were awake. This may be due to the absence of a siesta, which gave the *CASK-β* mutant flies a bigger time window during the day to feed (Figures 4D and 6G).

## Discussion

We report here the development of a method for continuously following the exact location of many individual moving objects for several days. This technique promises to provide valuable data to researchers conducting long-term behavioral studies. The ability to capture movement within precise locations at high temporal resolution allows for more rigorous investigations into the detailed behavior of an animal subject. While there is no complete substitute for the experimenter directly observing an animal (whether live or from video), it is impractical for studies that involve weeks-long trials with many animals. Tracking software also has the benefit of not being subject to human scoring error or bias. The 50% FBL *CASK-β* data analyzed by the Tracker detected movements of as little as 5 pixels in any direction at a distance of ∼300 mm. That level of resolution detection (movement onset/stop, turn, spin, etc.) using human scoring was impossible.

The utility of higher and more accurate data throughput was demonstrated here by comparison of the DAM and Tracker program sleep analysis for the *CASK-β* mutant fly line. This mutant has a known locomotor phenotype [19] that was not evident using conventional DAM beam-cross analysis, but was detected using our tracking system. In fact, it is common for manipulations of neurons and genes involved in the regulation of sleep (e.g. components of dopaminergic, circadian, GABAergic, peptidergic and other subsystems) to have profound effects on locomotor output. Most of the strong “sleep” mutants are very pleiotropic. Disrupted function of *Shaker* [23] and its regulators *Sleepless* [24] and *Hyperkinetic* [23], dopaminergic signaling genes such as *DopR1* [15] and *fumin* [25], and stress and immunity response genes like *relish* [26], *BiP* [27], and *Hsp83* [28] all lead to complex behavioral phenotypes. Unfortunately, the sparse and position-biased data brought in by incident recording can lead to misinterpretations or misrepresentations of these complex effects.

The recent increase in tracking applications in circadian/sleep research is a testament to the desire of investigators to acquire more quantitative and qualitative information about fly activity. The Pysolo analysis suite and EthoVision (Noldus, Netherlands) record locomotor data using image capture techniques, with varying success [18], [20], [29]. These, along with the short-term behavior recorders cited earlier, are continuous to the point of being able to record data from still images/video or are effective for high-resolution short-term recording. While this provides more data than incident recording, low temporal resolutions still miss movement. For example, analysis of our Tracker data showed that switching the capture resolution from 1 sec to 5 sec resulted in missing 13% of the total distance traveled (1 fly tracked for 24 h, data not shown). In addition, short-term image-based tracking is focused on minute movements of a population of flies, and is unable to record manageable data beyond several minutes [13]. In contrast, the Tracker program is amenable to short-duration style data capture with the duration of long-term experiments, and unlike commercially available software packages, it is a public domain solution available for free download.

The use of direct tracking techniques has the potential to expand our current understanding of classic circadian and sleep mutants as well as known locomotion-defective mutants. By being able to detect phenotypes that were previously unknown, our basic understanding of sleep and locomotion could be fundamentally changed. For example, we showed here that locomotion and place preference were altered in *CASK-β* mutant flies at particular times of day, effects that were undetectable by DAM analysis, and that could not be observed with existing short-term locomotor capture programs. Indeed, the phenotype of the *CASK-β* mutant highlights the need to rigorously follow the behavioral state of the flies when measuring sleep. DAM analysis could not differentiate between reduced sleep and wakefulness with low levels of locomotion. It overestimated sleep in the mutant flies, and the crude assessment of locomotor activity greatly understated the locomotor differences between the two lines to the point of showing no difference at all (Figure 3F).

The tracking program used for our experiments was designed to track objects of any shape, in enclosures of any size/shape, with high temporal resolution and the capability for long-term recording. Thus, this system functions as a multipurpose data-collecting tool, producing very generic, yet diversely accessible data. While we have extracted several different locomotor parameters as examples (Figure 4), raw Tracker data are amenable to many different types of analyses that can be designed by the investigator and implemented in Matlab or whatever other processing program the user favors.

The ability of the Tracker program to capture location data provides an important new tool for understanding the relationship between sleep and other behaviors. With our wild type control flies, for example, we confirmed the sleep locations previously reported by Hendricks et al. [4] and Zimmerman et al. [18], showing that flies prefer to sleep near their food but not directly on it (Figure 6). Closer proximity to the food was seen only during active periods and is consistent with the idea that this is when the majority of feeding is taking place. More generally, however, the ability to monitor location as a function of sleep/wake state could be used to address a variety of behavioral questions. If the experiment can be designed to have location report the behavior in question (e.g. the proximity to a tethered female as a measure of courtship or a choice between two food depots as a measure of preference or learning), Tracker can provide insight into complex behavioral interactions. Although in this study we have used the Tracker program to assess movement associated with sleep and circadian rhythms in fruit flies, this methodology is extremely flexible, allowing the investigator to change test animals, arena type, or behavioral paradigm without rewriting the data capture program or analysis scripts. Therefore, we believe that our Tracker program system could be an important analysis tool in a wide range of behavioral studies.
