# Correction: High-Resolution Positional Tracking for Long-Term Analysis of *Drosophila* Sleep and Locomotion Using the “Tracker” Program

**DOI:** 10.1371/annotation/4c62d454-931e-4c48-841a-a701cb658a1c

**Published:** 2012-08-08

**Authors:** Nathan C. Donelson, Eugene Z. Kim, Justin B. Slawson, Christopher G. Vecsey, Robert Huber, Leslie C. Griffith

The first author's name was spelled incorrectly. The correct name is Nathan C. Donelson. The correct citation is: Donelson NC, Kim EZ, Slawson JB, Vecsey CG, Huber R, et al. (2012) High-Resolution Positional Tracking for Long-Term Analysis of Drosophila Sleep and Locomotion Using the “Tracker” Program. PLoS ONE 7(5): e37250. doi:10.1371/journal.pone.0037250 

